# The Comprehensive Analysis Illustrates the Role of CDCA5 in Breast Cancer: An Effective Diagnosis and Prognosis Biomarker

**DOI:** 10.1155/2023/7150141

**Published:** 2023-05-30

**Authors:** Yang Gao, Shuting Liu, Junyuan Yang, Min Su, Jingjing Xu, Hua Wang, Jingwei Zhang

**Affiliations:** ^1^Department of Gynecological Oncology, Zhongnan Hospital of Wuhan University, Wuhan, China; ^2^Hubei Key Laboratory of Tumor Biological Behaviors, Wuhan, China; ^3^Hubei Cancer Clinical Study Center, Wuhan, China; ^4^Department of Breast and Thyroid Surgery, Zhongnan Hospital of Wuhan University, Wuhan, Hubei, China

## Abstract

**Background:**

Several studies have been conducted to investigate the role of cell division cycle-associated 5 (CDCA5) in cancer. Its role in breast cancer, however, remains unknown.

**Methods:**

The Gene Expression Omnibus and Cancer Genome Atlas Program databases provided the open-access information needed for the research. The CCK8 and colony formation assays were used to measure cell proliferation. The capacity of breast cancer cells to invade and migrate was assessed using the transwell assay.

**Results:**

In our study, CDCA5 was identified as the interested gene through a series of bioinformatics analysis. We found a higher CDCA5 expression level in tissue and cells of breast cancer. Meanwhile, CDCA5 has been linked to increased proliferation, invasion, and migration of breast cancer cells, which was also associated with worse clinical features. The biochemical pathways, in which CDCA5 was engaged, were identified using biological enrichment analysis. Immune infiltration research revealed that CDCA5 was linked to enhanced activity of several immune function terms. Meanwhile, DNA methylation might be responsible for the aberrant level of CDCA5 in tumor tissue. In addition, CDCA5 could significantly increase the paclitaxel and docetaxel sensitivity, indicating that it has the potential for clinical application. Also, we found that CDCA5 is mainly localized in cell nucleoplasm. Moreover, in the breast cancer microenvironment, we found that CDCA5 is mainly expressed in malignant cells, proliferation T cells, and neutrophils.

**Conclusion:**

Overall, our findings suggest that CDCA5 is a potential prognostic indicator and target for breast cancer, which can indicate the direction of the relevant research.

## 1. Introduction

Breast cancer is the most frequent malignant tumor in women worldwide, accounting for around 1.7 million new cases and 500,000 deaths each year [1]. Breast cancer is classified into three types based on histological characteristics: endocrine-dependent breast cancer, erb-b2 receptor tyrosine kinase 2 (HER2)-positive breast cancer, and triple-negative breast cancer (TNBC) [2]. Early detection and surgery are the foundations of good therapy, with a 5-year survival rate of over 98% for localized breast cancer [3]. However, the 5-year survival rate for individuals at the advanced stage is only 30% [3]. As a result, in addition to new treatment options, effective diagnosis and prognosis biomarkers with the potential to be novel therapeutic targets are urgently needed.

The protein cell division cycle-associated 5 (CDCA5) regulates sister chromatid cohesion and segregation [4]. CDCA5 supports sister chromatid cohesion and correct cell chromosomal separation during meiosis and mitosis by maintaining the cohesive complex, which is critical in DNA repair [5]. Additionally, CDCA5 may influence the activity of cell cycle-associated proteins and transcription factors, influencing cancer cell proliferation and death [6]. For instance, Fu et al. discovered that CDCA5 may control the cell cycle, the Phosphoinositide 3-kinase (PI3K)/v-akt murine thymoma viral oncogene homolog 1 (AKT)/mechanistic target of rapamycin (mTOR) pathway, and dysregulated mitochondria-mediated apoptosis to operate as a tumor promoter in bladder cancer [7]. Zhang et al. discovered that CDCA5 might accelerate the growth of gastric cancer by increasing cyclin E1 expression [8]. Luo et al. revealed that speckle-type POZ protein might stimulate CDCA5 degradation in prostate cancer, hence controlling AKT pathway activity and disease development [9]. Moreover, Chen et al. discovered that CDCA5 was transcribed by E2F1 and might aid in the growth of liver cancer cells via the AKT pathway [10]. Shen et al. discovered that CDCA5 was overexpressed in pancreatic cancer and enhanced disease development by activating the ERK signaling pathway in vitro and in vivo investigations [11]. However, few studies have examined the function of CDCA5 in breast cancer up to this point.

In our research, CDCA5 was identified as the interested gene through a series of bioinformatics analysis. The findings demonstrate that CDCA5 was associated with more severe clinical characteristics and a worse prognosis. CDCA5 was found to increase the proliferation, invasion, and migration of breast cancer cells in subsequent studies. The aberrant expression of CDCA5 in tumor tissue may be caused by a high degree of DNA methylation. Moreover, we found a significant correlation between CDCA5 and the chemosensitivity of paclitaxel and docetaxel. Also, we found that CDCA5 is mainly localized in cell nucleoplasm. Moreover, in the breast cancer microenvironment, we found that CDCA5 is mainly expressed in malignant cells, proliferation T cells, and neutrophils.

## 2. Methods

### 2.1. Acquisition and Analysis of Public Data

The Gene Expression Omnibus (GEO) and Cancer Genome Atlas (TCGA) databases were two of the open-access datasets used in this investigation. In detail, the transcription profile (TCGA-BRCA; Data Category: transcriptome profiling; Data Type: Gene Expression Quantification; Workflow: HTSeq-Fragments Per Kilobase Million (FPKM)) and clinical information (TCGA-BRCA; Data Category: clinical; Data Format: bcr xml) of breast cancer patients in TCGA were downloaded through TCGA-GDC. After that, the FPKM file was converted to Transcripts Per Kilobase Million format. The datasets of GSE70947 and GSE100534 were obtained from the GEO database. The platform of GSE70947 was GPL13607 (Agilent-028004 SurePrint G3 Human GE 8x60K Microarray), containing 195 paired breast adenocarcinoma and adjacent normal tissue. The platform of GSE100534 was GPL6244 (Affymetrix Human Gene 1.0 ST Array). The annotation of TCGA data was conducted using the human genomic reference file Homo_sapiens.GRCh38.gtf. Before analysis, all of the data were preprocessed and standardized. R software was used to analyze differentially expressed genes (DEGs) using the “limma” package. Using the R software's “survival” package, clinical correlation and survival analyses were conducted. The STRING database (https://cn.string-db.org/) was used to create a protein–protein interaction (PPI) network. To assess the prediction effectiveness of dichotomized events, the receiver operating characteristic (ROC) curve was used. The Human Protein Atlas (HPA, https://www.proteinatlas.org/) database was used to get immunohistochemistry and subcellular localization images of CDCA5 in breast cancer tumors and normal tissue [12]. The sequencing data of cancer cell lines were downloaded and analyzed from Broad Institute Cancer Cell Line Encyclopedia (CCLE) database [13].

### 2.2. Analysis of Pathway Enrichment

To investigate the biological difference across CDCA5 low and high patients, pathway enrichment analysis was done using the Gene Set Enrichment Analysis (GSEA) algorithm [14]. Hallmark, Gene Ontology (GO), and the Kyoto Encyclopedia of Genes and Genomes (KEGG) were used as reference route sets.

### 2.3. Immune-Related Analysis

Several methods were used to quantify immune cells in the tumor microenvironment, including EPIC, CIBERSORT, MCPCOUNTER, QUANTISEQ, and TIMER [15–18]. The quantification of immune function terms was conducted using the single sample GSEA (ssGSEA) algorithm [19].

### 2.4. DNA Methylation and Drug Sensitivity Analysis

The EWAS Data Hub (https://ngdc.cncb.ac.cn/ewas/datahub/index) and the MEXPRESS database (https://mexpress.be/) were used to assess CDCA5 DNA methylation levels [20]. Based on the Genomics of Drug Sensitivity in Cancer (GDSC), drug sensitivity analysis was carried out [21].

### 2.5. Single-Cell Analysis of CDCA5

The Tumor Immune Single-Cell Hub 2 (TISCH2) project developed by Sun et al. was used to directly explore the single-cell expression of CDCA5 in breast cancer tissue [22].

### 2.6. Cell Lines and Quantitative Real-Time PCR

Human normal breast epithelial cell lines (MCF-10A) and breast cancer cell lines (T47D, MCF-7, MDA-MB-231, and MDA-MB-469) were obtained from the Cell Bank of Culture of the Chinese Academy of Sciences. All of the cells were cultured under standard conditions. An RNA simple Total RNA Kit (Tiangen, China) was used for total RNA, which was then reverse transcribed to cDNA. A Polymerase Chain Reaction (PCR) reaction system with a capacity of 20 liters was used. SyBr Green PCR system was used for PCR detection. The following primers were used for Quantitative Real-time Polymerase Chain Reaction (qRT-PCR): CDCA5, forward, 5′-GTCCAATCACCTCGCAGGA-3′; reverse, 5′-CGTCCAGCTCTCCTTCCTTG-3; GAPDH, forward, 5′-CATGGGTGTGAACCATGAGA-3′; reverse, 5′-CAGTGATGGCATGGACTGTG-3′.

### 2.7. Cell Transfection

Following the methodology and prior investigations, cell transfection was performed using the lipofectamine 2000 reagent. Shanghai GenePharma provided the control and sh-CDCA5 plasmids, which had the following target sequences: shRNA1, 5′-CGAGCATCCTCCCTGAAAT-3′; shRNA2, 5′-GGTCCCAGCTGTCCAATCA-3′; shRNA3, 5′-GCGGAAATCAGGCTCTGAA-3′.

### 2.8. CCK8 Assay

The CCK8 test was carried out using a CCK8 kit (Dojindo, Shanghai, China) following the protocol and prior investigations [23].

### 2.9. Colony Formation Assay

The colony formation experiment was performed using a standardized procedure with 500 cells per well [23].

### 2.10. Transwell Assay

Transwell assay was performed to evaluate the invasion and migration ability of cancer cells in a 24-plate well, which was based on the standardized process [23]. Corning transwell chambers with 8 *μ*m pore size were used for the transwell assay.

### 2.11. Statistical Analysis

For all statistical analyses, GraphPad Prism 8 and R software were utilized. *P*-values were two-sided, and statistical significance was defined as less than 0.05. The Kruskal–Wallis test was used for variables having a non-normal distribution, whereas the student's *T*-test was used to compare variables with a normal distribution.

## 3. Results

### 3.1. The Identification Process of CDCA5 as the Interested Gene in Our Study

**Figure 1:**
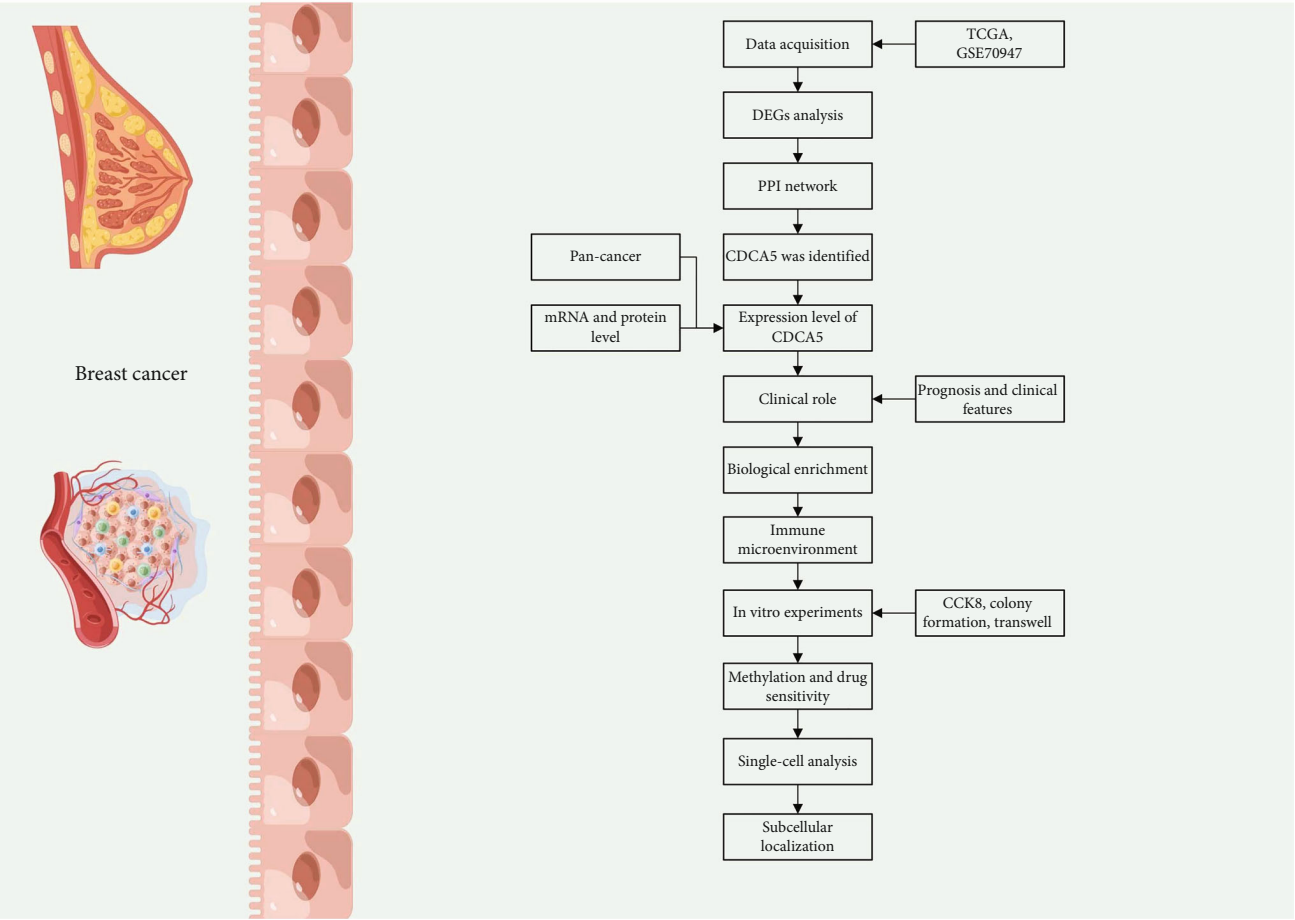
The flow chart of whole study.

**Figure 2:**
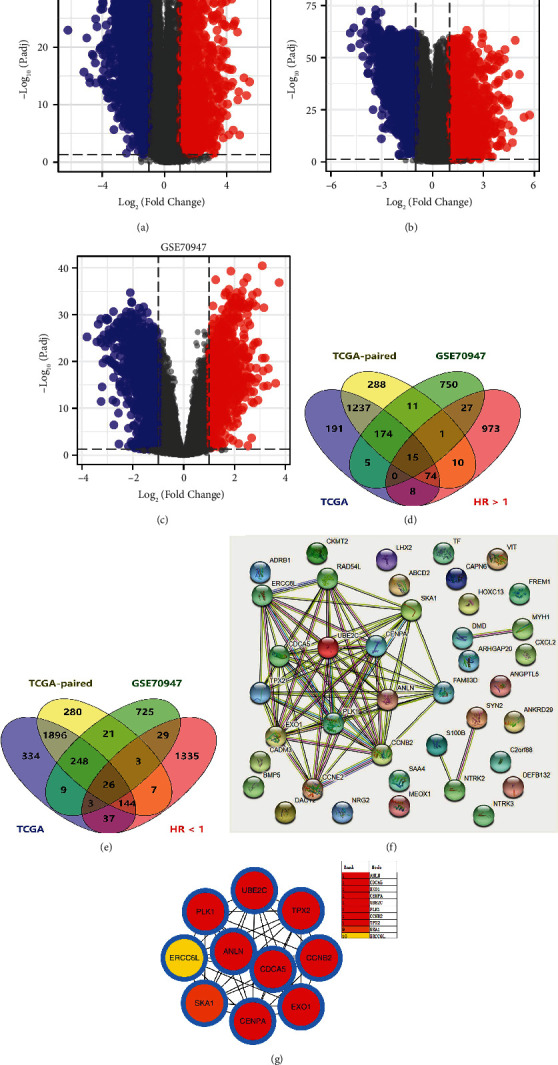
Identification of CDCA5 as the interested gene. (a) DEGs analysis between breast cancer and adjacent normal tissue (TCGA). (b) DEGs analysis between breast cancer and adjacent normal tissue (TCGA-paired). (c) DEGs analysis between breast cancer and adjacent normal tissue (GSE70947). (d) 15 genes were risk factors and commonly upregulated in TCGA and GSE70947. (e) 26 genes were protective factors and commonly downregulated in TCGA and GSE70947. (f) A PPI network of the above 41 genes. (g) The top 10 important nodes of the PPI network.

The flow chart of this study was presented in Figure 1. First, we analyzed DEGs in breast cancer and surrounding normal tissue using TCGA and GSE70947 data (Figures 2(a), 2(b), and 2(c)). Meanwhile, we found that 15 genes were risk factors and commonly upregulated in TCGA and GSE70947; 26 genes were protective factors and commonly downregulated in TCGA and GSE70947 (Figures 2(d) and 2(e)). We built a PPI network based on about 41 genes using the STRING database (Figure 2(f)). The top 10 significant nodes were shown in Figure 2(g). Of these nodes, CDCA5 was the second significant node and has not been fully explored in breast cancer, therefore arousing our interest (Figure 2(g)).

### 3.2. CDCA5 Was Upregulated in Breast Cancer Tissue and Might Be a Diagnosis Biomarker

**Figure 3:**
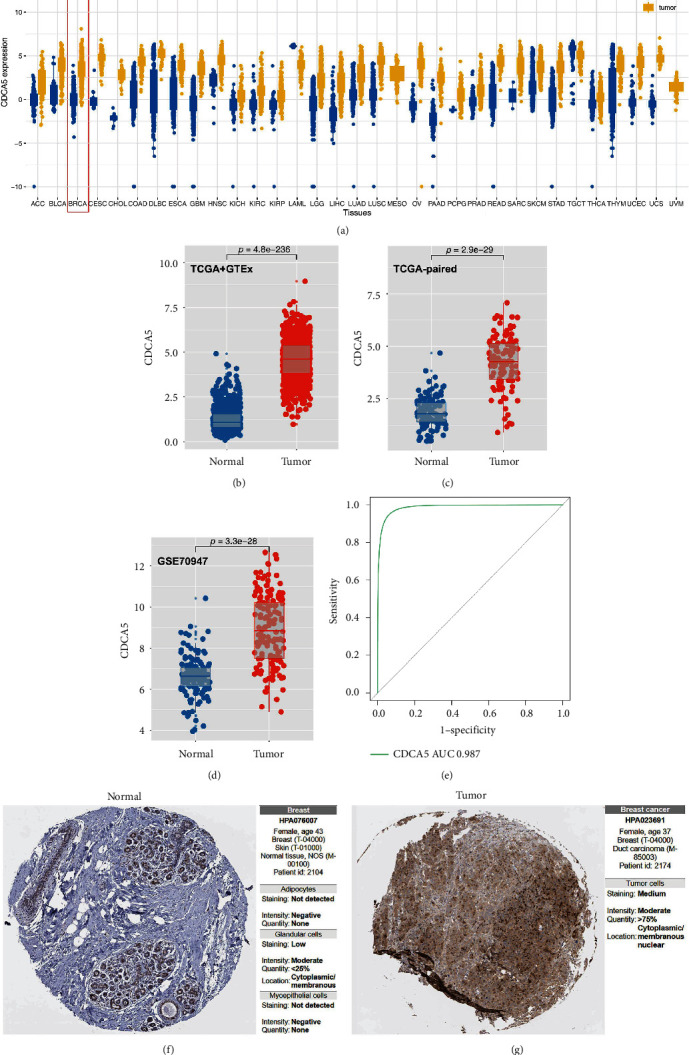
Expression pattern of CDCA5 in breast cancer. (a) Pan-cancer expression of CDCA5. (b) The expression level of CDCA5 in breast cancer and adjacent normal tissue (TCGA). (c) The expression level of CDCA5 in breast cancer and adjacent normal tissue (TCGA-paired). (d) The expression level of CDCA5 in breast cancer and adjacent normal tissue (GSE70947). (e) ROC curve indicate that CDCA5 has an extremely great prediction efficacy on breast cancer carcinogenesis. (f and g) The protein level of CDCA5 based on the IHC images of HPA database.

Figure 3(a) depicts the CDCA5 expression pattern in pan-cancer. We noticed an aberrant expression level between tumor and normal tissue in most cancer, indicating that CDCA5 has a potent cancer-related effect. In multiple breast cancer cohorts, a higher expression level was observed in breast cancer tissue (Figures 3(b), 3(c), and 3(d)). Moreover, we performed a ROC curve analysis to assess the prediction efficacy of CDCA5 on breast cancer. The result showed that the area under the curve (AUC) value was 0.987, indicating that CDCA5 has an extremely great prediction efficacy on breast cancer carcinogenesis (Figure 3(e)). Meanwhile, we obtained the immunocytochemistry image to assess the protein level of CDCA5 in breast cancer and normal tissue. The findings demonstrated that breast cancer tissue had a greater CDCA5 protein level than normal tissue (Figures 3(f) and 3(g)). Also, we found a diverse expression pattern of CDCA5 in different subtypes of breast cancer, including luminal, HER2-enriched, basal-like, and normal-like breast cancer (Figure S1).

### 3.3. CDCA5 Was Linked to Poorer Clinical Characteristics and a Poor Prognosis in Breast Cancer

**Figure 4:**
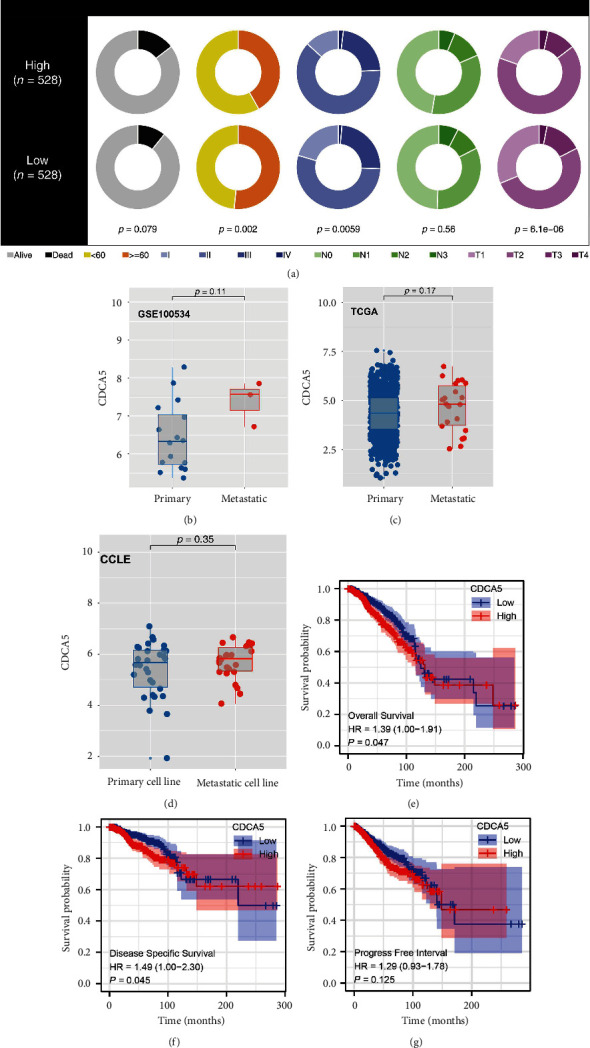
Clinical role of CDCA5 in breast cancer. (a) The difference of clinical parameters in patients with high and low CDCA5 expression. (b) The expression level of CDCA5 in primary and metastatic breast cancer tissue (GSE100534). (c) The expression level of CDCA5 in primary and metastatic breast cancer tissue (TCGA). (d) The expression level of CDCA5 in primary and metastatic breast cancer cell lines (CCLE). (e) OS difference in patients with high and low CDCA5 expression. (f) DSS difference in patients with high and low CDCA5 expression. (g) PFS difference in patients with high and low CDCA5 expression.

CDCA5 was related to poorer clinical-stage, T, and N classifications, according to clinical correlation analyses (Figure 4(a)). Interestingly, the younger breast patients (<60) seem to have a higher CDCA5 expression (Figure 4(a)). We investigated the CDCA5 level in primary and metastatic breast cancer tissue further. However, there is no significant difference in the expression of CDCA5 between metastatic and primary breast cancer tissues, which may be due to sample bias (Figures 4(b) and 4(c)). Considering the sample count bias, we think the *P*-value with statistically significant is inevitable. At the cell line level, no remarkable difference was observed in primary and metastatic cell lines (Figure 4(d)). Additionally, a Kaplan–Meier survival curve analysis was done to investigate the difference in prognosis between CDCA5 low and high patients. The result showed that the patients with high CDCA5 levels tend to have poor overall survival (OS) and disease specific survival (DSS) (Figures 4(e) and 4(f); OS, *P* = 0.047; DSS, *P* = 0.045). The *P*-value was not statistically significant in PFI (Figure 4(g)). However, a clear demarcation was observed in the 0–100 months of PFI survival curves between low and high CDCA5 patients, indicating that CDCA5 might affect disease progression in a specific period (Figure 4(g)).

### 3.4. Biological Enrichment Analysis of CDCA5 in Breast Cancer

**Figure 5:**
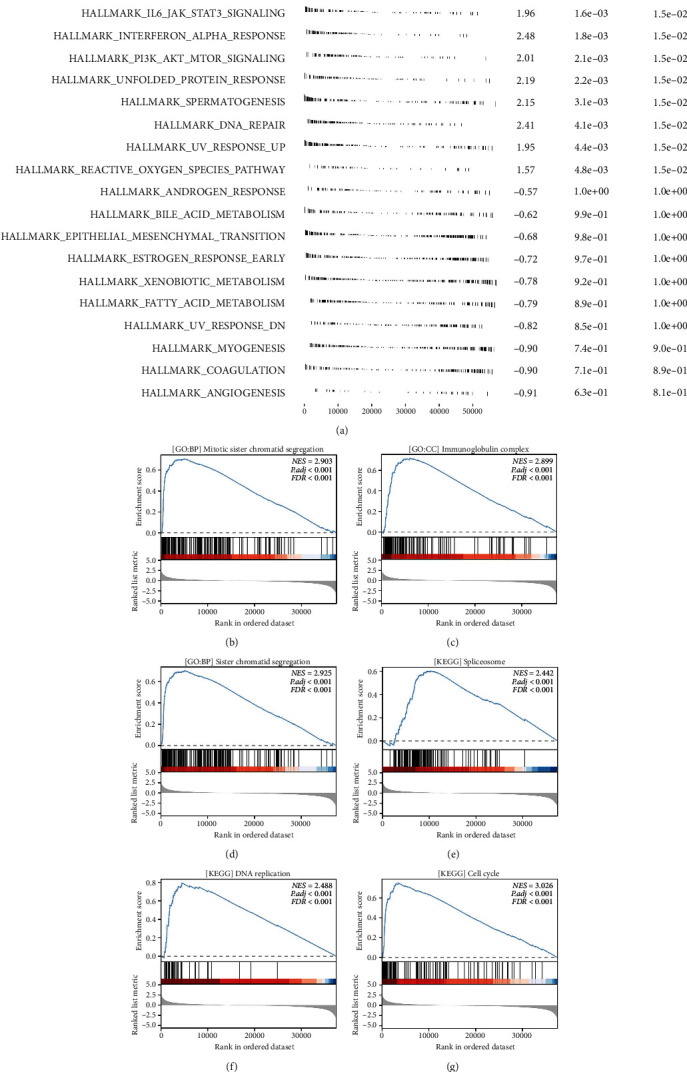
Biological enrichment pathway. (a) GSEA analysis of CDCA5 in breast cancer (Hallmark). (b, c, and d) GSEA analysis of CDCA5 in breast cancer (GO). (e, f, and g) GSEA analysis of CDCA5 in breast cancer (KEGG).

Subsequently, we attempted to investigate the underlying biological function of CDCA5 in breast cancer. We discovered that the pathways of v-myc avian myelocytomatosis viral oncogene homolog (MYC) targets, cholesterol homeostasis, interleukin 6 (IL6)/janus kinase (JAK)/signal transducer and activator of transcription 3 (STAT3) signaling, interferon (IFN) alpha response, and PI3L/AKT/mTOR signaling were considerably enriched in the GSEA analysis (Hallmark) (Figure 5(a)). The GSEA analysis based on the GO pathway set revealed that the top three pathways, in which CDCA5 was engaged, were mitotic sister chromatid segregation, immunoglobulin complex, and sister chromatid segregation (Figures 5(b), 5(c), and 5(d)). Based on the KEGG pathway set, the GSEA analysis revealed that the top three terms, in which CDCA5 was engaged, were spliceosome, DNA replication, and cell cycle (Figures 5(e), 5(f), and 5(g)).

### 3.5. Immune-Related Analysis

**Figure 6:**
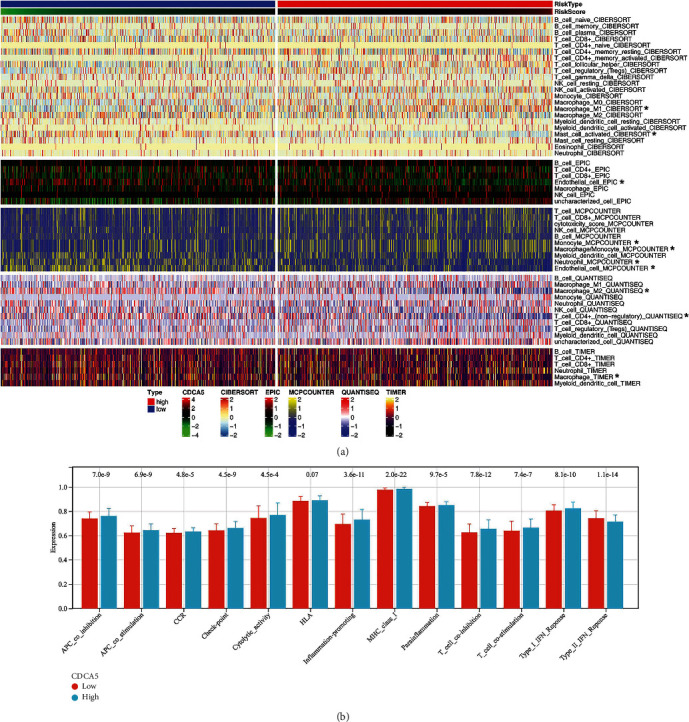
Immune-related analysis of CDCA5. (a) Quantification of immune cells based on multiple algorithms, including CIBERSORT, EPIC, MCPCOUNTER, QUANTISEQ, and TIMER. (b) Immune function difference in patients with high and low CDCA5 expression.

Based on the measured immune cells, we discovered that patients with high CDCA5 expression may have a larger level of monocyte and M1 macrophage infiltration, as well as a lower level of endothelial cells, activated mast cells, and CD4+ T cells (Figure 6(a)). Immune function study revealed that individuals with high CDCA5 expression were more active in APC_co_inhibition, APC_co_stimulation, chemokine (C-C motif) receptor, check-point, cytolytic activity, inflammation-promoting, MHC class I, para inflammation, T_cell_co-inhibition, T_cell_co-stimulation, and type I IFN response (Figure 6(b)).

### 3.6. CDCA5 Facilitates the Proliferation, Invasion, and Migration of Breast Cancer Cells

**Figure 7:**
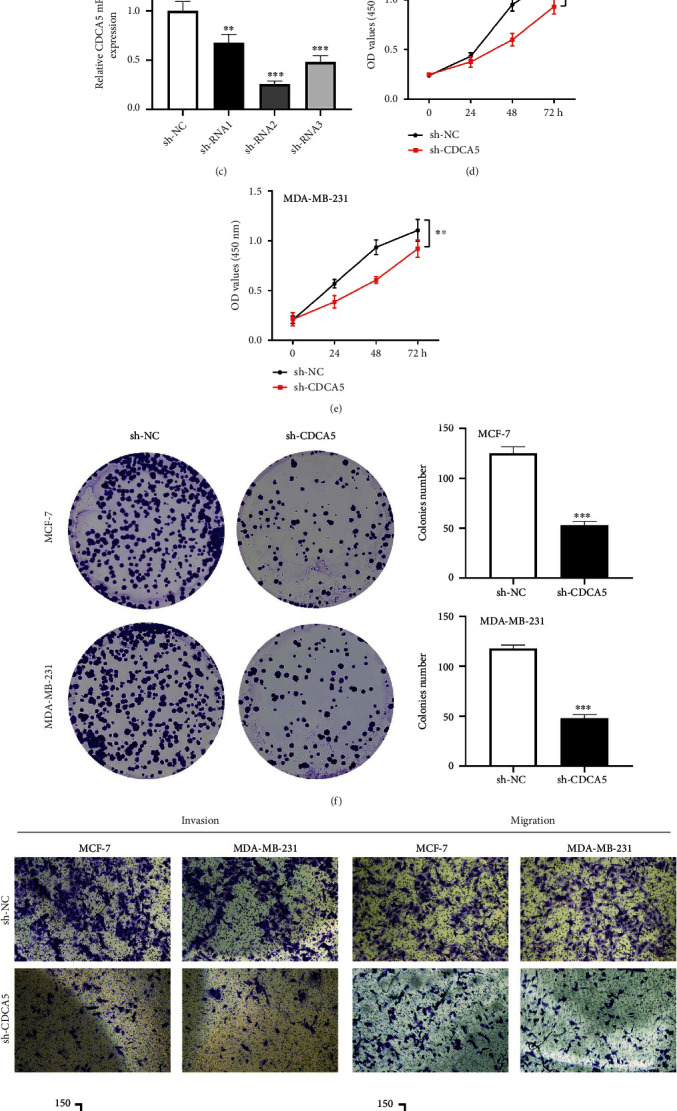
CDCA5 facilitates the proliferation, invasion, and migration of breast cancer cells. (a) The CDCA5 expression level in different breast cancer cell lines. (b and c) The knock down efficiency of CDCA5 in MCF-7 and MDA-MB-231 expression. (d and e) CCK assay was performed in sh-NC and sh-CDCA5 expression. (f) Colony formation assay was performed in sh-NC and sh-CDCA5 expression. (g) Transwell assay was performed in sh-NC and sh-CDCA5 expression.

Next, we conducted in vitro experiments to explore the biological role of CDCA5 at the cell level. CDCA5 was also discovered to be increased in breast cancer cell lines when compared to normal MCF-10A cells (Figure 7(a)). MCF-7 and MDA-MB-231 were chosen for additional testing because of their increased CDCA5 expression, and shRNA2 had the highest knockdown efficiency (Figures 7(b) and 7(c)). The CCK8 and colony formation assays revealed that the cells with CDCA5 inhibition might have a lower cell proliferation ability than the control cells (Figures 7(d), 7(e), and 7(f)). Transwell assays showed that the knockdown of CDCA5 might inhibit breast cancer cell invasion and migration ability (Figure 7(g)).

### 3.7. CDCA5 Might Be Regulated by DNA Methylation Level and Could Affect Chemosensitivity

**Figure 8:**
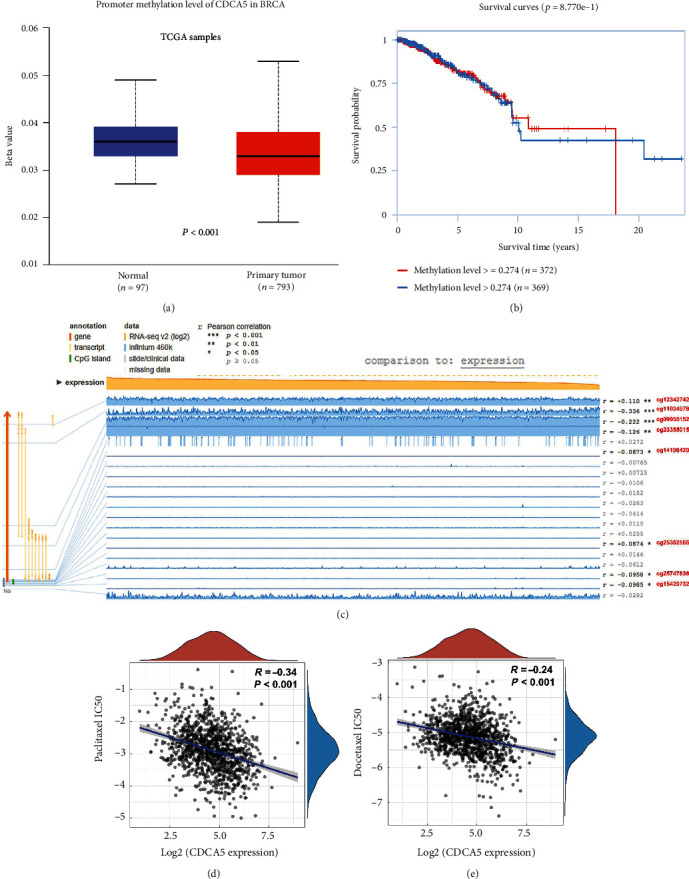
CDCA5 was regulated by DNA methylation level and could affect chemosensitivity. (a) Primary breast cancer tissue had a lower DNA methylation level of CDCA5 compared with the normal tissue. (b) Patients with low CDCA5 methylation level might have a worse prognosis. (c) The specific methylation site associated with CDCA5 expression. (d-e) Drug sensitivity analysis of CDCA5.

We examine the DNA methylation level of CDCA5 in tumor and normal tissue in light of its aberrant expression pattern. The result showed that the primary breast cancer tissue had a lower DNA methylation level of CDCA5 compared with the normal tissue, which might partly explain the high CDCA5 expression in breast cancer (Figure 8(a)). In addition, the patients with low CDCA5 methylation levels might have a worse prognosis (Figure 8(b)). To further identify the specific methylation site associated with CDCA5 expression, we performed a correlation analysis based on the MEXPRESS database. The result showed that the site cg11034978 (*r* = −0.336), cg09955152 (*r* = −0.222), cg23355015 (*r* = −0.126), cg14198420 (*r* = −0.0873), cg25747838 (*r* = −0.0958), and cg15429732 (*r* = −0.0985) were negatively correlated with CDCA5 expression, yet the site cg12342742 (*r* = 0.110) and cg25382566 (*r* = 0.0874) were positively with CDCA5 expression (Figure 8(c)). The most often used chemotherapy regimens for breast cancer were paclitaxel and docetaxel. Thus, we further explore the underlying correlation between their chemosensitivity and CDCA5 expression. The result showed that CDCA5 could significantly increase the paclitaxel and docetaxel sensitivity (Figures 8(d) and 8(e); paclitaxel: *R* = −0.34, *P* < 0.001; docetaxel: *R* = −0.24, *P* < 0.001).

### 3.8. Analysis of CDCA5 at the Cell Level

Based on the result obtained from the HPA database, we noticed that CDCA5 is mainly localized in cell nucleoplasm (Figures S2(a), S2(b), and S2(c), A-431 and U2OS cell lines). Moreover, in the breast cancer microenvironment, we found that CDCA5 is mainly expressed in malignant cells, proliferation T cells, and neutrophils (Figures S3(a), S3(b), and S3(c)).

## 4. Discussion

Breast cancer remains a threat worldwide for its high incidences [24]. Nowadays, for advanced breast cancer, molecular target therapy is integral for modern breast cancer treatment. As a result, it is critical to develop a unique and effective molecular target capable of guiding breast cancer detection and treatment.

The development of bioinformatics can provide direction for finding potential biological targets [25–28]. Here, CDCA5 was identified as the interested gene through a series of bioinformatics analysis. The findings demonstrate that CDCA5 was associated with more severe clinical characteristics and a worse prognosis. Moreover, we found that CDCA5 was upregulated in breast cancer cell lines, especially in MCF-7 and MDA-MB-231. Therefore, the MCF-7 and MDA-MB-231 were selected for further experiments. Then, CDCA5 was found to increase the proliferation, invasion, and migration of breast cancer cells in subsequent studies. The aberrant expression of CDCA5 in tumor tissue may be caused by a high degree of DNA methylation. Moreover, we found a significant correlation between CDCA5 and the chemosensitivity of paclitaxel and docetaxel. Also, we found that CDCA5 is mainly localized in cell nucleoplasm. Moreover, in the breast cancer microenvironment, we found that CDCA5 is mainly expressed in malignant cells, proliferation T cells, and neutrophils.

The CDCA5 gene encodes a protein involved in mitosis and plays a crucial function in the cell cycle [29]. Abnormal division cycles are commonly observed in cancer cells [30]. The CDCA5 gene encodes a protein involved in mitosis and plays a crucial function in the cell cycle. Gao et al. discovered that miR-326 adversely regulates CDCA5 and may accelerate ovarian cancer growth [31]. Xu et al. discovered that CDCA5 is a cancer–testis gene that promotes the proliferation, invasion, and migration of esophageal squamous cell carcinoma [32]. Chong et al. found that inhibiting CDCA5 might dramatically reduce the malignant biological activity of prostate cancer cells [33]. CDCA5 was shown to be elevated in breast cancer tissue and cell lines in our investigation. Meanwhile, the AUC value of CDCA5 on breast cancer prediction was 0.987, indicating that CDCA5 was an effective early diagnostic biomarker. Early identification is critical for breast cancer treatment, and the 5-year survival rate for early-stage patients is about 98% [3]. Meanwhile, a remarkable association was observed between CDCA5 and worse clinical features. CDCA5 was shown to greatly increase the proliferation, invasion, and migration of breast cancer cells in vitro. These results indicated that CDCA5 might be a novel diagnosis and therapeutic molecular marker with the potential for clinical application.

GSEA analysis revealed that in breast cancer patients with elevated CDCA5 expression, the PI3K/AKT/mTOR pathway was abnormally activated. Earlier research has also shown that CDCA5 might influence cell malignant behavior in prostate cancer, bladder cancer, and hepatocellular carcinoma through modulating PI3K/AKT/mTOR signaling [7, 9, 10]. PI3K/AKT/mTOR signaling has been extensively demonstrated to play an oncogenic function in many malignancies as a typical signaling pathway [34]. The stimulation of PI3K/AKT/mTOR signaling boosts cancer cells' capacity to grow, survive, and adapt metabolically, making it an attractive therapeutic target in cancer [35]. Several medicines targeting PI3K/AKT/mTOR signaling in breast cancer have reached the pre-clinical development stage [36]. Meanwhile, PI3K/AKT/mTOR signaling inhibition is required for the anti-tumor activity of HER2-targeted treatment, as well as mediating resistance to anti-HER2 therapy [37]. Moreover, some studies have shown that PI3K inhibitors could affect the treatment effects of endocrine therapy [38].

Paclitaxel and docetaxel were the common chemotherapy regimen in breast cancer therapy, and the resistance to them is still a medical issue of great concern. Researchers have begun to explore the mechanism at the biological level. Chang et al. found that mitochondrial transplantation could regulate the anti-tumor activity, chemoresistance (doxorubicin/paclitaxel), and mitochondrial dynamics in breast cancer [39]. Wu et al. also showed that LINC00160 might mediate paclitaxel/doxorubicin resistance in breast cancer cells by recruiting CCAAT/enhancer binding protein *β* to the trefoil factor 3 (intestinal) promoter [40]. Cazet et al. conducted a clinical experiment that demonstrated that targeting stromal remodeling and cancer stem cell plasticity might overcome docetaxel chemoresistance in TNBC [41]. Yan et al. discovered that the fibrous sheath interacting protein 1 protein promotes breast cancer growth and imparts resistance to docetaxel via stabilizing Recombinant Multidrug Resistance Associated Protein 1 [42]. CDCA5 was found to dramatically boost the paclitaxel and docetaxel sensitivity of breast cancer patients in drug sensitivity studies. This result indicated that for the patients with higher CDCA5 levels, paclitaxel and docetaxel might have a better therapeutic effect.

To summarize, this is the first study that thoroughly investigated the role of CDCA5 in breast cancer. CDCA5 expression was shown to be greater in breast cancer tissue and cells. Meanwhile, CDCA5 has been linked to increased proliferation, invasion, and migration of breast cancer cells, as well as inferior clinical outcomes. GSEA analysis was performed to determine the biological pathways in which CDCA5 was implicated. Immune infiltration research revealed that CDCA5 was linked to enhanced activity of several immune function terms, including monocyte and macrophages. Jiang et al. demonstrated that monocytes/macrophages can enhance the progression of breast cancer, which was regulated by CD137 [43]. Meanwhile, DNA methylation might be responsible for the aberrant level of CDCA5 in tumor tissue. In addition, CDCA5 could significantly increase the paclitaxel and docetaxel sensitivity, indicating that it has the potential for clinical application.

## Data Availability

All data can be obtained from the corresponding author based on reasonable request.

## Supplementary Materials

Supplementary Materials Figure S1. The expression level of CDCA5 in different subtypes of breast cancer. Figure S2. The subcellular localization of CDCA5 in cells. (a) The subcellular localization of CDCA5 in A-431 cells. (b) The subcellular localization of CDCA5 in U2OS cells. (c) CDCA5 was mainly localized in cell nucleoplasm. Figure S3. Single-cell analysis of CDCA5 in breast cancer microenvironment. (a) Single-cell analysis of CDCA5 in EMTAB8107 cohort. (b) Single-cell analysis of CDCA5 in GSE114727 cohort. (c) Single-cell analysis of CDCA5 in Alxe cohort.
