# Supplementary material for: The Comprehensive Analysis Illustrates the Role of CDCA5 in Breast Cancer: An Effective Diagnosis and Prognosis Biomarker
Source: Int J Genomics. 2023 May 30;2023:7150141. doi: 10.1155/2023/7150141 (PMC10243952; doi:10.1155/2023/7150141)
Supplement: Supplementary Materials — Figure S1. The expression level of CDCA5 in different subtypes of breast cancer. Figure S2. The subcellular localization of CDCA5 in cells. (a) The subcellular localization of CDCA5 in A-431 cells. (b) The subcellular localization of CDCA5 in U2OS cells. (c) CDCA5 was mainly localized in cell nucleoplasm. Figure S3. Single-cell analysis of CDCA5 in breast cancer microenvironment. (a) Single-cell analysis of CDCA5 in EMTAB8107 cohort. (b) Single-cell analysis of CDCA5 in GSE114727 cohort. (c) Single-cell analysis of CDCA5 in Alxe cohort. [file 7150141.f1.pdf]

Supplementary file

Figure S1

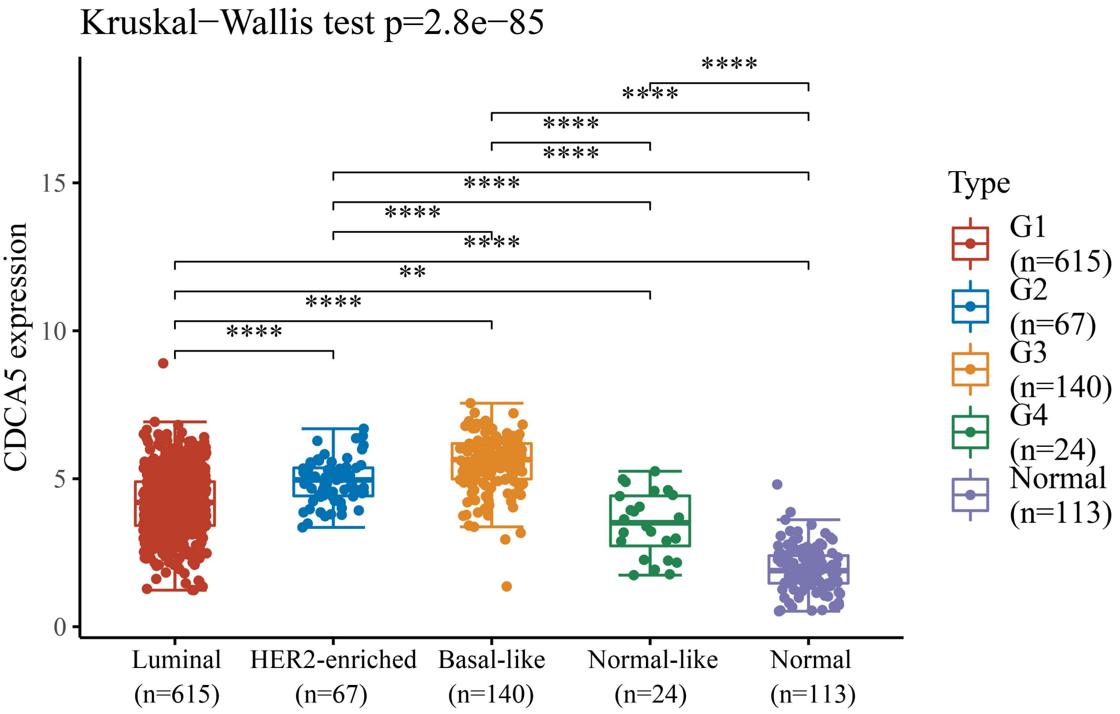

Figure S2

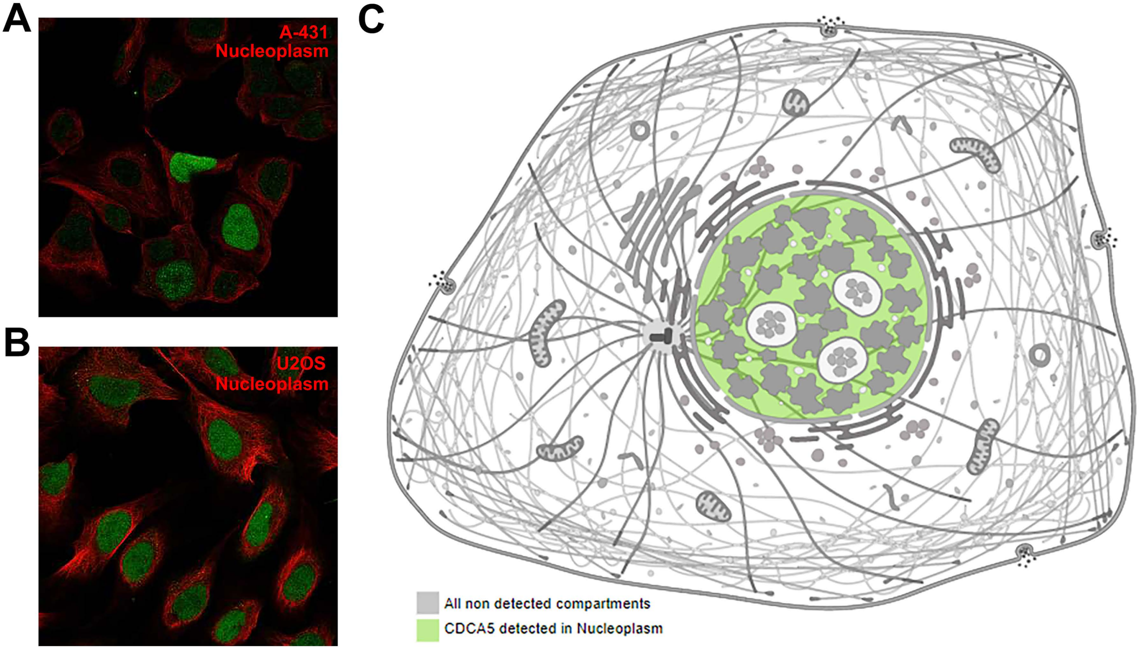
A
A-431
Nucleoplasm
B
U2OS
Nucleoplasm
C
All non detected compartments
CDCA5 detected in Nucleoplasm

Figure S3

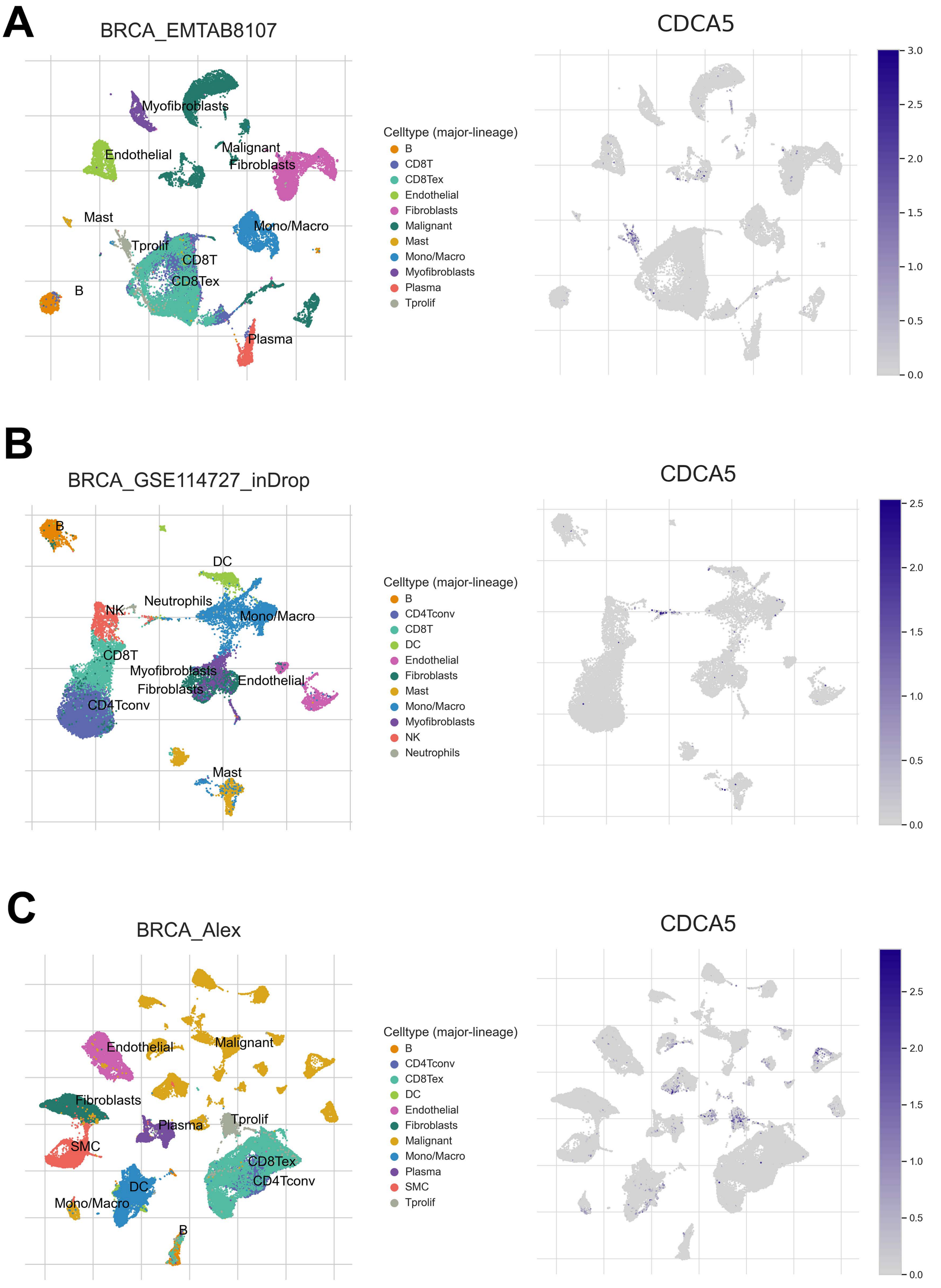

A
BRCA_EMTAB8107
Myofibroblasts
Endothelial
Malignant
Fibroblasts
Mast
Mono/Macro
Tprolif
CD8T
CD8Tex
B
Plasma
Celltype (major-lineage)
B
CD8T
CD8Tex
Endothelial
Fibroblasts
Malignant
Mast
Mono/Macro
Myofibroblasts
Plasma
Tprolif
CDCA5
3.0
2.5
2.0
1.5
1.0
0.5
0.0
B
BRCA_GSE114727_inDrop
B
DC
Neutrophils
NK
Mono/Macro
CD8T
Myofibroblasts
Fibroblasts
Endothelial
CD4Tconv
Mast
Celltype (major-lineage)
B
CD4Tconv
CD8T
DC
Endothelial
Fibroblasts
Mast
Mono/Macro
Myofibroblasts
NK
Neutrophils
CDCA5
2.5
2.0
1.5
1.0
0.5
0.0
C
BRCA_Alex
Endothelial
Malignant
Fibroblasts
Tprolif
Plasma
SMC
CD8Tex
CD4Tconv
DC
Mono/Macro
B
Celltype (major-lineage)
B
CD4Tconv
CD8Tex
DC
Endothelial
Fibroblasts
Malignant
Mono/Macro
Plasma
SMC
Tprolif
CDCA5
2.5
2.0
1.5
1.0
0.5
0.0
